# 5,6-Dimethyl-1*H*-benzimidazol-3-ium nitrate

**DOI:** 10.1107/S1600536813027578

**Published:** 2013-10-16

**Authors:** Bao-Cheng Liu, Shou-Jin Zhu, Fa-Qian Liu

**Affiliations:** a Key Laboratory of Advanced Materials, Qingdao University of Science and Technology, Qingdao 266042, People’s Republic of China

## Abstract

The title salt, C_9_H_11_N_2_^+^·NO_3_^−^, features a planar cation (r.m.s. for 11 non-H atoms = 0.016 Å). In the crystal, N—H⋯O hydrogen bonds link nitrate and benzimidazole ions into a three-dimensional network.

## Related literature

For background to benzimidazole, see: Roderick *et al.* (1972 ▶). For related crystal structures, see: Lee & Scheidt (1986 ▶), Liu (2012 ▶), Cui *et al.* (2009 ▶).
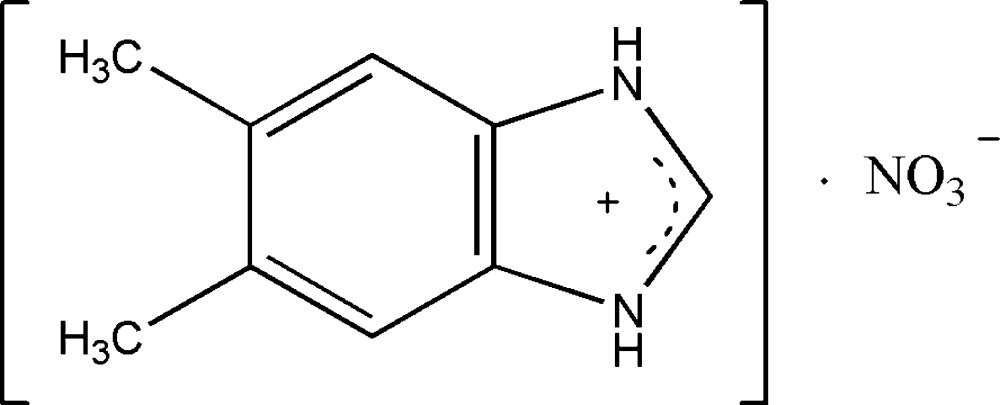


## Experimental

### 

#### Crystal data

C_9_H_11_N_2_^+^·NO_3_^−^
*M* *_r_* = 209.21
Monoclinic, 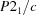

*a* = 6.938 (4) Å
*b* = 14.694 (8) Å
*c* = 10.379 (6) Å
β = 108.598 (9)°
*V* = 1002.8 (10) Å^3^
*Z* = 4
Mo *K*α radiation
μ = 0.11 mm^−1^
*T* = 296 K
0.29 × 0.27 × 0.22 mm

#### Data collection

Rigaku R-AXIS Spider diffractometer
Absorption correction: multi-scan (*ABSCOR*; Higashi 1995 ▶) *T*_min_ = 0.970, *T*_max_ = 0.977
5401 measured reflections
1973 independent reflections
1617 reflections with *I* > 2σ(*I*)
*R*_int_ = 0.028

#### Refinement

*R*[*F* ^2^ > 2σ(*F* ^2^)] = 0.045
*wR*(*F* ^2^) = 0.136
*S* = 1.05
1973 reflections
145 parameters
H atoms treated by a mixture of independent and constrained refinement
Δρ_max_ = 0.18 e Å^−3^
Δρ_min_ = −0.16 e Å^−3^

### 

Data collection: *RAPID-AUTO* (Rigaku, 2004 ▶); cell refinement: *RAPID-AUTO*; data reduction: *RAPID-AUTO*; program(s) used to solve structure: *SHELXS97* (Sheldrick, 2008 ▶); program(s) used to refine structure: *SHELXL97* (Sheldrick, 2008 ▶); molecular graphics: *SHELXTL* (Sheldrick, 2008 ▶); software used to prepare material for publication: *SHELXTL*.

## Supplementary Material

Crystal structure: contains datablock(s) global, I. DOI: 10.1107/S1600536813027578/hg5349sup1.cif

Structure factors: contains datablock(s) I. DOI: 10.1107/S1600536813027578/hg5349Isup2.hkl

Supplementary material file. DOI: 10.1107/S1600536813027578/hg5349Isup3.cdx

Supplementary material file. DOI: 10.1107/S1600536813027578/hg5349Isup4.cml

Additional supplementary materials:  crystallographic information; 3D view; checkCIF report

## Figures and Tables

**Table 1:** Hydrogen-bond geometry (Å, °)

*D*—H⋯*A*	*D*—H	H⋯*A*	*D*⋯*A*	*D*—H⋯*A*
N2—H2⋯O1^i^	0.96 (3)	2.31 (3)	3.043 (3)	133.0 (15)
N2—H2⋯O3^i^	0.96 (3)	1.86 (3)	2.797 (3)	165 (2)
N1—H1⋯O1^ii^	0.90 (3)	2.60 (2)	3.191 (3)	123.8 (17)
N1—H1⋯O2^ii^	0.90 (3)	1.89 (2)	2.797 (3)	178 (2)

Symmetry codes: (i) 
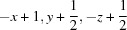
; (ii) 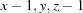.

**Fig. 1.**
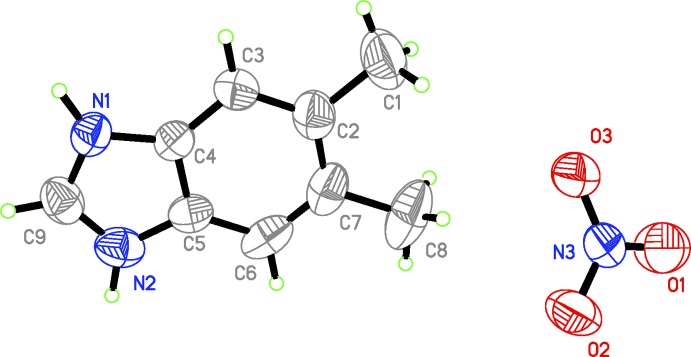
The structure of the title compound, showing 50% probability displacement ellipsoids and the atom-numbering scheme.

**Fig. 2.**
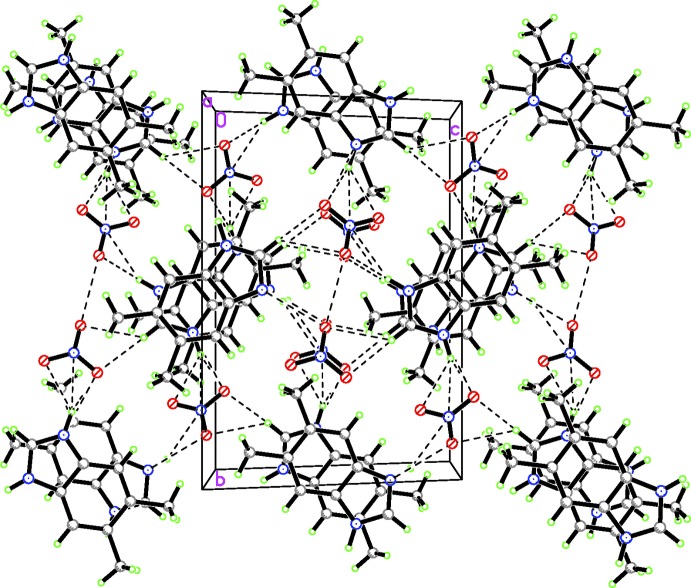
The crystal packing diagram viewed down the a axis.

## supplementary crystallographic information

### 1. Comment

Benzimidazole and its derivatives have attracted increased interest, not only because of their biological activity, but their abilities to bind to different metal ions (Roderick *et al.*, 1972). In this paper, we describe the synthesis and structure of the title compound C_9_H_11_N_3_O_3_. In the title compound the molecules are linked by N—H···O hydrogen bonds between nitrate and benzimidazole ions into a three-dimensional network structure. Some 5,6-dimethylbenzimidazole derivatives with similar structures have been reported, which include 5.6-Dimethylbenzimidazole (Lee & Scheidt, 1986), 5,6-dimethyl-lH-benzo[*d*]imidazol-3-ium 2-(4-chlorophenoxy)acetate (Liu, 2012),and Bis(5,6-dicarboxybenzimidazolium) sulfate monohydrate (Cui *et al.*, 2009).

### 2. Experimental

A mixture of 5,6-Dimethylbenzimidazole (2.86 mg, 0.02 mmol) and Co(NO_3_)_2_.6H_2_O (5.82 mg, 0.02 mmol) was added to H_2_O (20 ml). The mixture was refluxed for half an hour then filtered. The resulting solution was allowed to stand at room temperature to give yellow block crystals suitable for structural determination after 3 weeks. Analysis, calculated for C_9_H_11_N_3_O_3_: C 51.67, H 5.30, N 20.09%; Found: C 51.61, H 5.25, N 20.19%.

### 3. Refinement

H atoms on N1 and N2 atoms were positioned geometrically and allowed to ride on their parent atoms with N—H = 0.90 or 0.96 Å. H atoms of the methyl groups were positioned geometrically (C—H = 0.96 Å) and allowed to ride on their parent atoms with U_iso_(H) = 1.5 times U_eq_(C). All the other H atoms were positioned geometrically(C—H = 0.93 Å) and allowed to ride on their parent atoms with U_iso_(H) = 1.2 times U_eq_(C).

### Crystal data

**Table:**

C_9_H_11_N_2_^+^·NO_3_^−^	*F*(000) = 440
*M**_r_* = 209.21	*D*_x_ = 1.386 Mg m^−^^3^
Monoclinic, *P*2_1_/*c*	Mo *K*α radiation, λ = 0.71073 Å
Hall symbol: -P 2ybc	Cell parameters from 3271 reflections
*a* = 6.938 (4) Å	θ = 2.5–26.6°
*b* = 14.694 (8) Å	µ = 0.11 mm^−^^1^
*c* = 10.379 (6) Å	*T* = 296 K
β = 108.598 (9)°	Block, yellow
*V* = 1002.8 (10) Å^3^	0.29 × 0.27 × 0.22 mm
*Z* = 4	

### Data collection

**Table:**

Rigaku R-AXIS Spider diffractometer	1617 reflections with *I* > 2σ(*I*)
Radiation source: fine-focus sealed tube	*R*_int_ = 0.028
Graphite monochromator	θ_max_ = 26.0°, θ_min_ = 2.5°
ω scans	*h* = −8→8
Absorption correction: multi-scan (*ABSCOR*; Higashi 1995)	*k* = −17→18
*T*_min_ = 0.970, *T*_max_ = 0.977	*l* = −8→12
5401 measured reflections	13 standard reflections every 0 reflections
1973 independent reflections	intensity decay: none

### Refinement

**Table:**

Refinement on *F*^2^	Secondary atom site location: difference Fourier map
Least-squares matrix: full	Hydrogen site location: inferred from neighbouring sites
*R*[*F*^2^ > 2σ(*F*^2^)] = 0.045	H atoms treated by a mixture of independent and constrained refinement
*wR*(*F*^2^) = 0.136	*w* = 1/[σ^2^(*F*_o_^2^) + (0.0764*P*)^2^ + 0.1519*P*] where *P* = (*F*_o_^2^ + 2*F*_c_^2^)/3
*S* = 1.05	(Δ/σ)_max_ < 0.001
1973 reflections	Δρ_max_ = 0.18 e Å^−^^3^
145 parameters	Δρ_min_ = −0.16 e Å^−^^3^
0 restraints	Extinction correction: *SHELXL97* (Sheldrick, 2008), Fc^*^=kFc[1+0.001xFc^2^λ^3^/sin(2θ)]^-1/4^
Primary atom site location: structure-invariant direct methods	Extinction coefficient: 0.102 (10)

### Special details

**Table:**

Geometry. All esds (except the esd in the dihedral angle between two l.s. planes) are estimated using the full covariance matrix. The cell esds are taken into account individually in the estimation of esds in distances, angles and torsion angles; correlations between esds in cell parameters are only used when they are defined by crystal symmetry. An approximate (isotropic) treatment of cell esds is used for estimating esds involving l.s. planes.
Refinement. Refinement of F^2^ against ALL reflections. The weighted R-factor wR and goodness of fit S are based on F^2^, conventional R-factors R are based on F, with F set to zero for negative F^2^. The threshold expression of F^2^ > 2sigma(F^2^) is used only for calculating R-factors(gt) etc. and is not relevant to the choice of reflections for refinement. R-factors based on F^2^ are statistically about twice as large as those based on F, and R- factors based on ALL data will be even larger.

### Fractional atomic coordinates and isotropic or equivalent isotropic displacement parameters (Å^2^)

**Table:**

	*x*	*y*	*z*	*U*_iso_*/*U*_eq_	
C6	0.3017 (3)	0.52774 (14)	0.13819 (18)	0.0638 (5)	
H6A	0.3096	0.5767	0.1965	0.077*	
C3	0.2734 (3)	0.38091 (11)	−0.03709 (17)	0.0545 (4)	
H3A	0.2625	0.3319	−0.0956	0.065*	
N3	0.9477 (2)	0.33163 (9)	0.55623 (14)	0.0615 (4)	
C4	0.2174 (2)	0.46728 (10)	−0.08774 (14)	0.0451 (4)	
C1	0.4099 (4)	0.27501 (17)	0.1557 (2)	0.0936 (8)	
H1A	0.3917	0.2333	0.0816	0.140*	
H1B	0.3287	0.2558	0.2102	0.140*	
H1C	0.5506	0.2761	0.2104	0.140*	
C2	0.3452 (3)	0.36885 (13)	0.10052 (19)	0.0613 (5)	
C8	0.4308 (4)	0.4293 (2)	0.3399 (2)	0.1049 (9)	
H8A	0.4287	0.4865	0.3839	0.157*	
H8B	0.5670	0.4058	0.3678	0.157*	
H8C	0.3432	0.3870	0.3647	0.157*	
C7	0.3576 (3)	0.44249 (15)	0.18821 (18)	0.0645 (5)	
C5	0.2327 (2)	0.53977 (10)	−0.00159 (17)	0.0493 (4)	
N2	0.1683 (2)	0.61491 (10)	−0.08221 (17)	0.0609 (4)	
C9	0.1169 (3)	0.58998 (12)	−0.20846 (19)	0.0602 (5)	
H9A	0.0678	0.6289	−0.2824	0.072*	
N1	0.1439 (2)	0.50169 (10)	−0.21736 (13)	0.0519 (4)	
O1	0.9388 (3)	0.31136 (10)	0.66814 (13)	0.0858 (5)	
O2	1.0253 (3)	0.40438 (9)	0.53833 (14)	0.0857 (5)	
O3	0.8828 (3)	0.27846 (9)	0.45932 (13)	0.0829 (5)	
H1	0.108 (3)	0.4713 (15)	−0.297 (2)	0.082 (7)*	
H2	0.156 (4)	0.6760 (18)	−0.053 (2)	0.097 (7)*	

### Atomic displacement parameters (Å^2^)

**Table:**

	*U*^11^	*U*^22^	*U*^33^	*U*^12^	*U*^13^	*U*^23^
C6	0.0503 (9)	0.0868 (13)	0.0550 (10)	−0.0109 (9)	0.0176 (7)	−0.0263 (9)
C3	0.0557 (9)	0.0498 (9)	0.0583 (9)	−0.0039 (7)	0.0187 (7)	−0.0015 (7)
N3	0.0792 (10)	0.0433 (7)	0.0533 (8)	0.0036 (7)	0.0090 (7)	0.0041 (6)
C4	0.0417 (8)	0.0517 (8)	0.0436 (7)	−0.0050 (6)	0.0161 (6)	−0.0024 (6)
C1	0.0874 (16)	0.0854 (15)	0.0949 (16)	−0.0108 (12)	0.0109 (12)	0.0373 (12)
C2	0.0511 (9)	0.0712 (11)	0.0599 (10)	−0.0090 (8)	0.0155 (7)	0.0135 (8)
C8	0.0874 (16)	0.175 (3)	0.0469 (11)	−0.0045 (17)	0.0142 (10)	0.0111 (14)
C7	0.0479 (9)	0.0968 (14)	0.0472 (9)	−0.0077 (9)	0.0128 (7)	0.0073 (9)
C5	0.0413 (8)	0.0518 (9)	0.0559 (9)	−0.0042 (6)	0.0172 (6)	−0.0079 (7)
N2	0.0529 (8)	0.0488 (8)	0.0779 (10)	0.0012 (6)	0.0163 (7)	−0.0064 (7)
C9	0.0499 (9)	0.0584 (10)	0.0699 (11)	0.0022 (7)	0.0156 (8)	0.0138 (8)
N1	0.0517 (8)	0.0601 (9)	0.0442 (7)	−0.0023 (6)	0.0157 (6)	−0.0004 (6)
O1	0.1216 (13)	0.0780 (9)	0.0593 (8)	−0.0232 (8)	0.0309 (8)	−0.0002 (7)
O2	0.1434 (14)	0.0470 (7)	0.0653 (9)	−0.0202 (7)	0.0315 (9)	0.0006 (6)
O3	0.1287 (13)	0.0498 (7)	0.0549 (7)	−0.0080 (7)	0.0078 (7)	−0.0012 (6)

### Geometric parameters (Å, º)

**Table:**

C6—C7	1.364 (3)	C1—H1B	0.9600
C6—C5	1.386 (3)	C1—H1C	0.9600
C6—H6A	0.9300	C2—C7	1.399 (3)
C3—C2	1.366 (3)	C8—C7	1.505 (3)
C3—C4	1.381 (2)	C8—H8A	0.9600
C3—H3A	0.9300	C8—H8B	0.9600
N3—O1	1.2197 (19)	C8—H8C	0.9600
N3—O2	1.237 (2)	C5—N2	1.371 (2)
N3—O3	1.2393 (19)	N2—C9	1.296 (2)
C4—C5	1.373 (2)	N2—H2	0.96 (3)
C4—N1	1.374 (2)	C9—N1	1.318 (2)
C1—C2	1.505 (3)	C9—H9A	0.9300
C1—H1A	0.9600	N1—H1	0.90 (2)
			
C7—C6—C5	118.47 (16)	C7—C8—H8A	109.5
C7—C6—H6A	120.8	C7—C8—H8B	109.5
C5—C6—H6A	120.8	H8A—C8—H8B	109.5
C2—C3—C4	118.80 (16)	C7—C8—H8C	109.5
C2—C3—H3A	120.6	H8A—C8—H8C	109.5
C4—C3—H3A	120.6	H8B—C8—H8C	109.5
O1—N3—O2	120.70 (15)	C6—C7—C2	120.78 (17)
O1—N3—O3	120.23 (15)	C6—C7—C8	118.5 (2)
O2—N3—O3	119.06 (15)	C2—C7—C8	120.7 (2)
C5—C4—N1	106.25 (15)	N2—C5—C4	106.54 (15)
C5—C4—C3	120.73 (15)	N2—C5—C6	132.67 (16)
N1—C4—C3	133.01 (14)	C4—C5—C6	120.79 (16)
C2—C1—H1A	109.5	C9—N2—C5	108.69 (15)
C2—C1—H1B	109.5	C9—N2—H2	124.2 (14)
H1A—C1—H1B	109.5	C5—N2—H2	127.1 (14)
C2—C1—H1C	109.5	N2—C9—N1	110.46 (16)
H1A—C1—H1C	109.5	N2—C9—H9A	124.8
H1B—C1—H1C	109.5	N1—C9—H9A	124.8
C3—C2—C7	120.41 (18)	C9—N1—C4	108.06 (14)
C3—C2—C1	118.79 (19)	C9—N1—H1	123.2 (14)
C7—C2—C1	120.80 (18)	C4—N1—H1	128.6 (14)
			
C2—C3—C4—C5	−0.2 (2)	C3—C4—C5—N2	179.37 (14)
C2—C3—C4—N1	179.02 (15)	N1—C4—C5—C6	179.55 (13)
C4—C3—C2—C7	1.3 (2)	C3—C4—C5—C6	−1.0 (2)
C4—C3—C2—C1	−178.89 (16)	C7—C6—C5—N2	−179.34 (16)
C5—C6—C7—C2	−0.1 (3)	C7—C6—C5—C4	1.2 (2)
C5—C6—C7—C8	−179.48 (16)	C4—C5—N2—C9	0.16 (18)
C3—C2—C7—C6	−1.1 (3)	C6—C5—N2—C9	−179.36 (17)
C1—C2—C7—C6	179.06 (18)	C5—N2—C9—N1	−0.22 (19)
C3—C2—C7—C8	178.23 (17)	N2—C9—N1—C4	0.19 (18)
C1—C2—C7—C8	−1.6 (3)	C5—C4—N1—C9	−0.09 (16)
N1—C4—C5—N2	−0.04 (16)	C3—C4—N1—C9	−179.40 (17)

### Hydrogen-bond geometry (Å, º)

**Table:**

*D*—H···*A*	*D*—H	H···*A*	*D*···*A*	*D*—H···*A*
N2—H2···O1^i^	0.96 (3)	2.31 (3)	3.043 (3)	133.0 (15)
N2—H2···O3^i^	0.96 (3)	1.86 (3)	2.797 (3)	165 (2)
N1—H1···O1^ii^	0.90 (3)	2.60 (2)	3.191 (3)	123.8 (17)
N1—H1···O2^ii^	0.90 (3)	1.89 (2)	2.797 (3)	178 (2)

Symmetry codes: (i) −*x*+1, *y*+1/2, −*z*+1/2; (ii) *x*−1, *y*, *z*−1.
